# It's More Than Complex: Further Insights Into TP53 in MPN

**DOI:** 10.1002/ajh.27632

**Published:** 2025-02-08

**Authors:** Nico Gagelmann, Nicolaus Kröger

**Affiliations:** ^1^ Department of Stem Cell Transplantation University Medical Center Hamburg‐Eppendorf Hamburg Germany

**Keywords:** leukemia, mpn, myelofibrosis, tp53

## Abstract

TP53 in MPN.

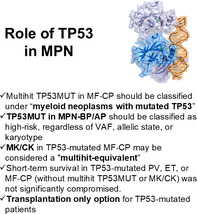

## 1 Editorial

TP53, the “guardian of the genome,” plays a critical role in cellular repair and apoptosis, and its mutations (TP53MUT) are among the most significant markers of poor prognosis in hematologic malignancies. While extensively studied in myelodysplastic neoplasms and acute myeloid leukemia [1, 2], TP53MUT in myeloproliferative neoplasms (MPNs) has received relatively less attention.

The study at hand by Tefferi and colleagues, examining 114 patients with TP53MUT across diverse MPN subtypes, provides a crucial lens into the prognostic and therapeutic implications of these mutations in MPNs [3].

### 1.1 Key Insights and Clinical Implications

First, the study confirms recent findings in myelofibrosis (MF) undergoing allogeneic hematopoietic cell transplantation (HCT) [4], underscoring that the prognostic impact of TP53MUT in MPNs depends significantly on its allelic configuration and associated karyotypic abnormalities. Multihit TP53MUT (present in 56% of cases) was strongly associated with poor survival, particularly in chronic‐phase MF (MF‐CP). In this context, multihit TP53MUT independently predicted worse outcomes, irrespective of other molecular risk factors, VAF, or karyotype. These findings solidify its role as a dominant driver of prognosis in MF‐CP [4, 5].

In advanced MPN subtypes, such as accelerated phase (MPN‐AP) and blast phase (MPN‐BP), the presence of TP53MUT was universally linked to dismal outcomes, with median survival of 4–6 months. However, in these aggressive subtypes, the allelic state of TP53MUT had less prognostic impact, suggesting that the stage of disease may overshadow the specific genetic configuration. In contrast, in earlier‐phase MPNs, such as PV and ET, the absence of multihit TP53MUT correlated with relatively favorable outcomes, though the long‐term impact remains uncertain.

A particularly thought‐provoking finding is the equivalency of monosomal and/or complex karyotype (MK/CK) to multihit TP53MUT in determining poor prognosis in MF‐CP. This observation aligns with recent studies in MDS and AML, reinforcing the idea that MK/CK may serve as a “multihit‐equivalent” marker in TP53‐mutated myeloid neoplasms. One caveat, particularly in the setting of advanced MF, is the limited quality of bone marrow analysis for karyotyping due to the massive fibrosis. However, these insights could inform future iterations of classification systems and risk models for MPNs.

### 1.2 Therapeutic Challenges and Future Directions

The therapeutic landscape for TP53MUT MPNs remains fraught with challenges. Allogeneic stem cell transplantation is the only curative option for MF [6]. The study reports a median post‐transplant survival of just 9 months in TP53MUT multi‐hit configuration, with relapse rates exceeding 50%. These results, despite being worse than what has been recently reported in another international HCT cohort in MF with 6‐year overall survival of 56% for individuals with single‐hit versus 25% for those with multi‐hit TP53MUT [4]. More evidence is needed on the role of HCT versus non‐HCT approaches in MF, although first signals suggest improved outcomes for HCT across TP53MUT groups [7].

Emerging therapies targeting TP53 or exploiting synthetic lethality mechanisms may hold promise. Advances in molecular profiling and targeted therapy development should prioritize this high‐risk patient population. Additionally, integrating TP53 allelic state and karyotype into treatment algorithms could enable more personalized approaches, optimizing outcomes while minimizing treatment‐related toxicity.

### 1.3 Broader Implications for Classification and Research

The study's findings resonate with evolving classification frameworks for myeloid neoplasms. The authors propose including TP53‐mutated MPN‐BP/AP and multi‐hit MF‐CP in the broader category of “myeloid neoplasms with mutated TP53.” [8, 9] This proposal aligns with established criteria for TP53‐mutated MDS and AML, where multi‐hit status confers distinct prognostic and biological significance.

## 2 Conclusion

This study represents a significant step forward in our understanding of TP53MUT in MPNs. It underscores the mutation's profound impact on prognosis, particularly in multihit configurations and adverse karyotypes, and highlights the limitations of current therapeutic strategies. As our molecular understanding of MPNs deepens, integrating TP53 mutation status into risk stratification and treatment paradigms will be crucial.

The findings also serve as a call to action for the scientific community. The poor outcomes associated with TP53MUT demand intensified efforts to develop novel personalized risk stratification, therapies and refine existing approaches.

Future research should explore the long‐term impact of TP53MUT in earlier‐phase MPNs, such as PV and ET, where its role remains less clear. Longitudinal studies incorporating ultra‐deep sequencing and single‐cell genomics could elucidate the clonal dynamics of TP53MUT and its interaction with other driver mutations. Such efforts would enhance our understanding of disease progression and resistance mechanisms, paving the way for more effective interventions.

By aligning research efforts with clinical insights, we can aspire to transform the grim prognosis of TP53‐mutated MPNs into a challenge surmountable through innovation and collaboration.

## Ethics Statement

The authors have nothing to report.

## Conflicts of Interest

The authors declare no conflicts of interest.

## Data Availability

The data that support the findings of this study are available by e‐mail request to the corresponding author.
